# Opportunities for primary health care in South Africa – Reflections from the USA

**DOI:** 10.4102/phcfm.v15i1.4250

**Published:** 2023-08-23

**Authors:** Robert J. Mash

**Affiliations:** 1 Division of Family Medicine and Primary Care, Faculty of Medicine and Health Sciences, Stellenbosch University, Cape Town, South Africa

In sub-Saharan Africa, we have a narrative about our weak primary health care systems – a lack of infrastructure, scarcity of healthcare workers and too few resources in terms of equipment, supplies and medication.^1^ However, a recent meeting at the National Academies of Science, Engineering and Medicine in the United States of America (USA) gave me a different perspective.^2^

At this meeting, it was blatantly stated that there is no high-quality primary health care system in the USA for most of the population. Primary care services are fragmented, disease-orientated and wedded to fee-for-service payments that do not promote comprehensive, continuous and coordinated care or whole person medicine.^3^ Among its peers in the Organisation for Economic Co-operation and Development (OECD), the USA is an outlier – spending a significantly higher percentage of gross domestic product (GDP) on health (17.8% vs. OECD average of 9.6%) and obtaining significantly lower life expectancy (77.0 years vs. OECD average of 80.4 years). The Commonwealth Fund’s Issue Brief states that ‘not only is the US the only country we studied that does not have universal health coverage, but its health system can seem designed to discourage people from using services’.^4^

One obvious explanation for this is the absence of effective high quality primary health care. Primary health care is acknowledged as the only part of the health system that improves health status, reduces costs and enhances health equity.^5^

This conference, on ‘The Essential Role of Primary Health Care for Health Security and Securing Health’, was organised by the Centre for Professionalism and Value in Health Care in Washington, D.C.^2^ The conference agenda was built on the 2021 US National Academies’ report ‘Implementing High Quality Primary Care’^3^ that set out five key goals:

- Pay for primary care teams to care for people, not doctors to deliver services.
- Ensure that high-quality primary care is available to every individual and family in every community.
- Train primary care teams where people live and work.
- Design information technology that serves the patient, family and interprofessional care team.
- Ensure that high-quality primary care is implemented in the United States.

The conference organisers had hoped that the Department of Health and Human Services (HSS) in the USA would have published their action plan on primary health care ahead of the meeting, but this is still eagerly awaited.

Discussions at the conference highlighted some of the major obstacles to achieving the goals. The health system is in the grip of private sector companies whose core value is making profit for shareholders. High-quality primary health care, with successful health promotion and disease prevention, would actually threaten these profits. One participant exclaimed that it is fundamentally un-American to not make a profit or see healthcare as a business. Others spoke about the difficulties of attempting to measure the core functions of primary care from data intended to monitor billing and fee-for-service payments. Despite Medicare, Medicaid, Obamacare (the *Affordable Care Act*) and other attempts to extend insurance cover, the USA has the highest rates of infant and maternal deaths in the OECD community and the lowest rates of physician visits and numbers of practising physicians.^4^ One is left with the impression that there is a battle of values with a commitment to individualism and profit, winning out over the common good.

Why then does this give me a different perspective on African health systems – and particularly the South African system which I know best? When I look at the South African health care system, I see that the accepted and proclaimed values at the heart of the system provide the foundation on which we can build high-quality primary health care. The public sector serves the majority of the population and is fundamentally committed to person-centred, community-orientated primary health care for all. These values are embodied in initiatives such as our district health system, national health insurance, the ideal clinic, ward-based outreach teams, the training of family physicians and community-orientated primary care.^6,7,8^

Yes, of course, we have many problems, such as diminishing health budgets, corruption and impunity of corrupt officials and poor managerial and leadership capacity, to name a few. However, from the vantage point of the USA, these appear to be easier problems to solve, so long as we hold on to our central values and strong commitment to a primary health care approach. The National Health Insurance White Paper in South Africa asserted our commitments to the right to access health care, social solidarity, equity, health care as a public good, affordability, efficiency, effectiveness and appropriateness.^9^ An uncontested system built on this foundation can lead to the expected results of health security, universal health coverage, improved determinants of health and achievement of the health-related sustainable development goals.^10^
